# Clinical impact of post-mortem genetic testing in cardiac death and cardiomyopathy

**DOI:** 10.1515/med-2020-0150

**Published:** 2020-05-19

**Authors:** Isabelle Marey, Véronique Fressart, Caroline Rambaud, Paul Fornes, Laurent Martin, Sarah Grotto, Yves Alembik, Hervé Gorka, Gilles Millat, Estelle Gandjbakhch, Céline Bordet, Geoffroy Lorin de la Grandmaison, Pascale Richard, Philippe Charron

**Affiliations:** APHP, Reference Center for Hereditary Heart Diseases, Department of Genetics, Pitié-Salpêtrière Hospital, 75013 Paris, France; APHP, Cardiogenetics and Myogenetics Unit, Metabolic Biochemistry Department, Pitié-Salpêtrière Hospital Group, 75013 Paris, France; Department of Pathology and Legal Medicine, Raymond Poincaré Hospital, APHP, UVSQ, 92380 Garches, France; Department of Pathology and Legal Medicine, Reims Hospital, 51100 Reims, France; Department of Pathology and Legal Medicine, Dijon Hospital, 21000 Dijon, France; Department of Medical Genetics, Robert Debré Hospital, 75019 Paris, France; Department of Medical Genetics, Strasbourg-Hautepierre Hospital, 67000 Strasbourg, France; Department of Cardiology, Chartres Hospital, 28000 Chartres, France; Molecular Cardiogenetics Laboratory, Center for Biology and Pathology East, Hospices Civils de Lyon, 69500 Bron, France; Sorbonne Université, INSERM, UMR_S 1166, ICAN Institute for Cardiometabolism and Nutrition, 75013 Paris, France

**Keywords:** sudden death, cardiac death, cardiomyopathy, post-mortem, genetic testing

## Abstract

Post-mortem genetic analyses may help to elucidate the cause of cardiac death. The added value is however unclear when a cardiac disease is already suspected or affirmed. Our aim was to study the feasibility and medical impact of post-mortem genetic analyses in suspected cardiomyopathy. We studied 35 patients with cardiac death and suspected cardiomyopathy based on autopsy or clinical data. After targeted sequencing, we identified 15 causal variants in 15 patients (yield 43%) in sarcomeric (*n* = 8), desmosomal (*n* = 3), lamin A/C (*n* = 3) and transthyretin (*n* = 1) genes. The results had various impacts on families, i.e. allowed predictive genetic testing in relatives (15 families), planned early therapeutics based on the specific underlying gene (5 families), rectified the suspected cardiomyopathy subtype (2 families), assessed the genetic origin of cardiomyopathy that usually has an acquired cause (1 family), assessed the diagnosis in a patient with uncertain borderline cardiomyopathy (1 family), reassured the siblings because of a *de novo* mutation (2 families) and allowed prenatal testing (1 family). Our findings suggest that post-mortem molecular testing should be included in the strategy of family care after cardiac death and suspected cardiomyopathy, since genetic findings provide additional information useful for relatives, which are beyond conventional autopsy.

## 1 Introduction

Cardiomyopathies are a heterogeneous group of heart muscle disorders defined by the presence of a structurally and functionally abnormal myocardium in the absence of coronary artery disease, hypertension, valvular disease and congenital heart disease sufficient to cause the observed myocardial abnormality [1,2]. The four major subtypes of cardiomyopathy are dilated cardiomyopathy (DCM), hypertrophic cardiomyopathy (HCM), restrictive cardiomyopathy (RCM) and arrhythmogenic right ventricular cardiomyopathy (ARVC) as well as unclassified cardiomyopathies such as left ventricular non-compaction (LVNC). All cardiomyopathies can be inherited [3,4,5,6], especially in an autosomal dominant mode in most cases. The underlying genes can be related to the genes encoding sarcomeric, cytoskeleton, desmosomal or other proteins, and a causal variant can usually be identified in 20–60% of patients with an obvious cardiomyopathy, depending on the subtypes and sequencing strategy [3,4,5,6,7].

Cardiomyopathies are estimated to be responsible for 10–35% of sudden cardiac deaths (SCDs) in the young population [8,9,10]. Other possible complications of cardiomyopathies include heart failure, stroke and thromboembolic complications. Because cardiac expression of inherited cardiomyopathies is frequently delayed, the disease is often clinically silent for a number of years and complications such as SCD can be the presenting feature [4,9,10,11]. Molecular genetic testing may be performed after cardiac death, especially after SCD and autopsy, and the guidelines promote the prescription of post-mortem genetic testing with a relatively high level of recommendation in the context of SCD and suspected channelopathy or cardiomyopathy [11,12,13,14]. The clinical impact is quite obvious in the context of unexplained SCD with normal autopsy since post-mortem genetic analysis may elucidate the cause of unexplained death [15], although interpretation of pathogenicity remains a challenging issue. However, the clinical impact of post-mortem genetic analyses in the context of suspected cardiomyopathy is less established. Very few publications have specifically studied post-mortem genetic analyses in suspected cardiomyopathies and they focused on the feasibility and yield of causal variant screening [16,17,18,19,20] but did not study the clinical impact of these results on the family. The level of evidence for supporting post-mortem genetic analyses in the context of cardiomyopathies is therefore low.

The goal of our study was to report our experience about the feasibility of post-mortem molecular testing in a group of structural heart diseases, cardiomyopathies, and to study the medical impact of the results on the families.

## 2 Materials and methods

### 2.1 Patients and tissue

In this retrospective study, we collected data of post-mortem genetic analyses performed at a single molecular laboratory from patients with (i) SCD or other cardiovascular cause of death (such as acute heart failure), (ii) suspected cardiomyopathy, based on either autopsy or clinical data (if cardiac examination was performed before death) and (iii) appropriate quality of DNA extraction. A cohort of 35 patients (from six hospitals in France) with such criteria was retrieved from our database from 2007 to 2014 (samples were sent during this period, just after death, to the Molecular Cardiogenetics Unit at Pitié-Salpêtrière Hospital and sequenced according to the practice of genetic diagnosis at that moment). It was before the use of high-throughput sequencing in diagnostic routine, so molecular screening was limited to focused analyses with Sanger sequencing.

Genomic DNA was extracted from autopsy of frozen tissues obtained from 17 patients (heart tissue *n* = 14, pulmonary artery *n* = 1 and liver *n* = 2), post-mortem frozen blood (6 patients) or post-mortem paraffin-embedded tissue (1 patient). In 11 patients, a clinical sample (blood and fibroblasts) was available from the intensive care unit. Autopsy was performed in 21 patients, highlighting the structural changes related to suspected or definitive cardiomyopathies.

The first step of forensic autopsy involved external examination of the corpse. Then it was open from the chin to the pubis and the organs were examined *in situ* before being removed. The heart was taken off together with both the lungs and its proceeding was done according to the “Guidelines for autopsy investigations of sudden cardiac death” [21]. Microscopic examination was done on a whole biventricular transversal section and on the walls of the pulmonary artery ejection chamber (right myocardium): at least ten myocardium samples were histologically examined for each case (hemalun–eosin–saffron staining). Additionally, myocardium and/or spleen or liver samples were frozen and stored at −20°C.

In all cases, molecular studies were performed after death. Written informed consent was obtained from the close relatives of the deceased, and molecular analyses were performed in order to improve the care of the family, in agreement with French law and French good practices documents.

The family of the deceased was advised according to our multidisciplinary management established between the departments of medical genetics, cardiology and forensic medicine. The first-degree relatives were invited for cardiological assessment, including a detailed physical examination, electrocardiogram and echocardiography. Then in the case of identification of a causal variant in the deceased, the relatives were informed especially when the result may have potential implications for the family.

## 3 Genetic analysis

Extraction and purification of the total DNA from frozen tissues were done after grinding using the Tissue Lyser LT and the DNeasy® Blood & Tissue kit (Qiagen, Venlo, Netherlands). For other samples (blood and fibroblasts), the extraction was performed on a QiaSymphony® (Qiagen). Sanger sequencing was done using the Big Dye Terminator v3.3 Cycle Sequencing kit and ABI3130 capillary electrophoresis system (Applied Biosystems, Waltham, USA). The extracted DNA was quantified with spectrophotometry (Nanodrop; Thermoscientific, Waltham, USA) and qualified by agarose gel migration. Sequencing targeted the genes involved in the various types of suspected cardiomyopathies: five major sarcomeric genes (*MYH7*, *MYBPC3*, *TNNT2*, *TNNI3* and *MYL2*) in HCM; three desmosomal genes (*PKP2*, *DSP*, and *DSG2*) in ARVC; *LMNA* and the five major sarcomeric genes in DCM; and *TTR* in amyloidosis. The entire coding sequences and intronic adjacent sequences of genes were systematically analysed in all patients. Chromatographs were analysed with the SeqScape software (AB®) and the variants were numbered according to their RefSeq transcripts (details in Section 5). Alamut (Interactive Biosoftware, North Seattle, WA, USA) was used for variants’ numbering and to determine the pathogenicity of the variants via its links to prediction software.

## 4 Interpretation of variants

Pathogenicity of variants was determined according to the current American College of Medical Genetics (ACMG) guidelines [22], which recommend classifying variants into five categories: pathogenic (class 5), likely pathogenic (class 4), uncertain significance (class 3), likely benign (class 2) or benign (class 1). The identified variants were first checked for their allele frequency as reported in the databases about populations of the same ethnic origin (http://gnomad.broadinstitute.org/). We evaluated the putative pathogenicity of each missense variant by using our knowledge of genes and proteins (the location of the variant in the gene and its impact on the resulting corresponding protein), *in silico* prediction tools (Polyphen-2, SIFT and Mutation taster) and SpliceSiteFinder like^®^, MaxEntScan^®^, NNSPLICE^®^, GeneSplicer^®^ and Human Splicing Finder^®^ for putative splicing variants. We then looked at the score for pathogenicity (combined annotation-dependent depletion [CADD] score) calculated on the basis of multiple algorithms (Phred-like score). This was followed by a careful review of the literature, available published segregation analyses and functional studies. Finally, we searched our local database to check whether the variant was already found in familial forms of cardiomyopathies and/or arrhythmias and validated as pathogenic with segregation analysis.

In practice, we considered as “pathogenic” (class 5) a variant with confirmed pathogenicity criteria and already proved to be responsible for cardiomyopathies or a novel nonsense variant with a frequency below 0.01%. We considered as “likely pathogenic” (class 4) the unpublished variants with a frequency below 0.01% and unknown in our database, located in a functional domain of the protein and with pathogenicity prediction tools in favour of a strong effect with a CADD score of >24. Only variants of classes 5 and 4 (certainly pathogenic or probably pathogenic) were reported in this study and were referred to as “causal variants”. Unfortunately, except in few families, no informative segregation analyses could be done because there was no additional relative with a cardiac phenotype already expressed.

## 5 Results

### 5.1 Patients

**Figure 1:**
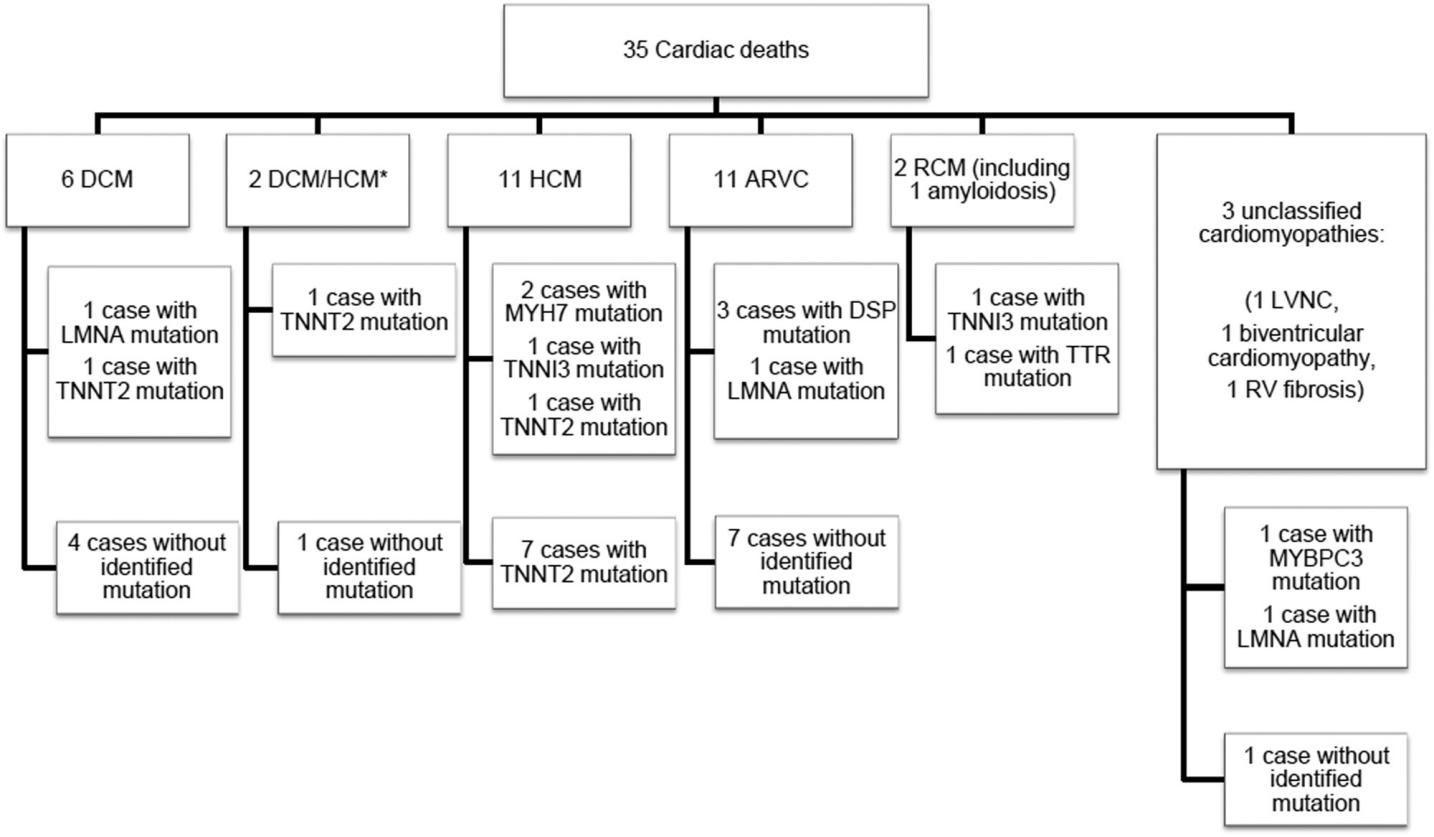
Overview of all patients according to the suspected cardiomyopathy. ARVC: arrhythmogenic right ventricular cardiomyopathy, HCM: hypertrophic cardiomyopathy, DCM: dilated cardiomyopathy, RCM: restrictive cardiomyopathy, LVNC: left ventricular non-compaction. *HCM/DCM: HCM observed in the deceased but DCM previously reported in a relative, subsequently interpreted as end-stage form of HCM.

Thirty-five patients were included in the study (mean age 25.5 years, age range: 21 days to 72 years, 18 males), with SCD (*n* = 28), death due to acute heart failure (*n* = 3), multiorgan failure (*n* = 2) or another vascular cause (cerebrovascular accident in 1 and pulmonary embolism in 1). Twenty-seven patients (77%) were under 40 years. Most patients were of Caucasian origin (26 of 35). Suspected cardiomyopathy was established after autopsy for 21 patients (in 17 patients the disease was also unknown within the family) and before death for 14 patients (6 with a diagnosis made in the intensive care unit and 8 with a previously known diagnosis). The suspected cardiomyopathy was HCM in 11, ARVC in 11, DCM in 6 patients, mixed HCM/DCM in 2, RCM in 2 (including cardiac amyloidosis in 1) and atypical cardiomyopathies in 3 (LVNC in 1, interstitial fibrosis without fibrofatty infiltration in 1 and biventricular cardiomyopathy in 1; Figure 1). Because of suspected cardiomyopathy, a cardiac evaluation was proposed to all first-degree relatives (in parallel to genetic molecular analyses in the deceased). In six families (20%), a systematic cardiac evaluation revealed borderline or obvious similar cardiomyopathy in at least one relative.

### 5.2 Post-mortem genetic results

Fifteen causal variants were identified in 15 patients (43% of the cohort; clinical features in Table 1): three causal variants in the *DSP* gene (desmoplakin), three in *LMNA* (lamin A/C), three in *TNNT2* (troponin T), two in *TNNI3* (troponin I), two in *MYH7* (beta myosin heavy chain 7), one in *MYBPC3* (myosin-binding protein C) and one in *TTR* (transthyretin). The detailed genetic results, NM numbers and main criteria for pathogenicity are detailed in Table 2. Considering the subtype of cardiomyopathies (Figure 1), a causal variant was identified in 4 of 11 HCM patients, in 4 of 11 ARVC patients, in 2 of 6 DCM patients, in the 2 RCM patients and in 1 of 2 patients with mixed HCM/DCM. Among the four variants classified as likely pathogenic, two were nonsense and one was a frameshift. All other variants were classified as certainly pathogenic.

**Table 1:** Clinical features of index patients with a causal variant after post-mortem analysis

Patients	Sex	Origin	Age of death	Context of death	Autopsy	Final clinical diagnosis of the disease	Family history
1	M	Turkey	35 years	Inaugural SCD	Fibrofatty replacement of right RV myocardium, HW: 430 g	ARVC	Supraventricular tachycardia in the mother
2	M	Egypt	32 years	Inaugural SCD	Fibrofatty replacement of both ventricles, HW 510 g	ARVC	SCD in one brother without precise diagnosis of the cause
3	M	France	33 years	Inaugural SCD	RV dilatation, fibrofatty replacement of RV, HW: 425 g	ARVC	SCD in the paternal uncle without precise diagnosis of the cause
4	F	France	39 years	SCD. ARVC known before death, with biventricular evolution	Not performed (post-mortem genetic analysis based on another tissue)	ARVC	None
5	M	The Netherlands	9 years	Inaugural SCD	Borderline for HCM (macroscopy: weight 230 g, septum thickness 7 mm, posterior wall 12 mm; histology: very small area with fibrosis and disarray)	HCM (suspected)	Suspected apical HCM in the mother (with peripartum heart failure)
6	F	France	52 years	Pulmonary embolism. HCM known before death	Not performed (post-mortem genetic analysis based on another tissue)	HCM	None
7	F	France	47 years	SCD. HCM known before death	Not performed (post-mortem genetic analysis based on another tissue)	HCM	HCM in the sister
8	M	Morocco	66 years	SCD. HCM known before death	Not performed (post-mortem genetic analysis based on another tissue)	HCM	SCD in two children (12 and 15 years), unknown cause, no autopsy
9	F	France	5 months	Acute heart failure/multiorgan failure and death. No clinical diagnosis before emergency management	No autopsy, but endomyocardial biopsy: subnormal histological result	LVNC	HCM in the grandmother (LV thickness 15 mm)
							HCM in the father (septum thickness 22 mm)
10	F	Algeria	16 years	Inaugural SCD	HCM at autopsy (LV septum thickness 28 mm, lateral wall 17 mm; microscopy: myocardial disarray)	HCM/DCM	DCM in the father (LVEF 30%, left atrium 61 mm)
11	F	France	2 years	Massive stroke. Diagnosis of RCM during emergency management	Not performed (but endomyocardial biopsy, non-specific findings)	RCM	None
12	M	France	44 years	Heart failure. DCM known before death	Not performed (post-mortem genetic analysis based on another tissue)	DCM	None
13	M	Morocco	1 month	Heart failure/multiorgan failure. No clinical diagnosis before emergency management	Not performed (post-mortem genetic analysis based on another tissue)	DCM	None
14	F	USA	74 years	Heart failure. RCM known before death.	Not performed (post-mortem genetic analysis based on another tissue)	RCM (amyloidosis)	Possible cardiac disease in brother (no detail)
15	M	France	59 years	SCD. DCM and AVB known before death.	Not performed (post-mortem genetic analysis based on another tissue)	Biventricular cardiomyopathy	None

ARVC: arrhythmogenic right ventricular cardiomyopathy; HCM: hypertrophic cardiomyopathy; DCM: dilated cardiomyopathy; RCM: restrictive cardiomyopathy; LVNC: left ventricular non-compaction; AVB: atrioventricular block; SCD: sudden cardiac death; LVEF: left ventricular ejection fraction; RV: right ventricle; HW: heart weight.

**Table 2:** Molecular genetic characteristics of causal variant identified after post-mortem analysis

Patients	Tissues for genetic tests	Gene	Variant	Variant	Frequency of variant (ALL)	Mutation taster	SIFT prediction	PolyPhen-2	CADD score	Previously reported variant in patients (HGMD) and phenotype	ACMG classification
		LRG_	(cDNA) level	LOVD_ID#0000229861	(GnomAD)						
1	Frozen tissue (autopsy)	*DSP*	c.[7027G>A]	p.(Glu2343Lys)	−	Disease-causing	Tolerated	Benign	24.5	Yes/ARVC	5
		LRG_423									

2	Frozen tissue (autopsy)	*DSP*	c.[790G>T]	p.(Glu264*)	−	−	−	−	39	No	4
		LRG_423									
3	Frozen tissue (autopsy)	*DSP*	c.[3367A>T]	p.(Arg1123*)	−	−	−	−	35	No	4
		LRG_423									
4	Sampling before death	*LMNA*	c.[1517_1520dup]	p.(Ser507Argfs*46)	−	−	−	−	−	No	4
		LRG_254									
5	Frozen tissue (autopsy)	*MYH7*	c.[2105T>A]	p.(Ile702Asn)	−	Disease-causing	Deleterious	Probably damaging	31	Yes/HCM	5
		LRG_384									
6	Sampling before death	*MYH7*	c.[1988G>A]	p.(Arg663His)	ALL: 0.0014%	Disease-causing	Damaging	Possibly damaging	27.8	Yes/HCM	5
		LRG_384									
7	Sampling before death	*TNNI3*	c.[407G>A]	p.(Arg136Gln)	ALL: 0.00040%	Disease-causing	Deleterious	Possibly damaging	28.2	Yes/HCM	5
		LRG_432									
8	Sampling before death	*TNNT2*	c.[275G>A]	p.(Arg92Gln)	−	Disease-causing	Deleterious	Probably damaging	35	Yes/HCM-LVNC	5
		LRG_431									
9	Sampling before death	*MYBPC3*	c.[2864_2865del]	p.(Pro955Argfs*95)	−	−	−	−		Yes/HCM	5
		LRG_386	+	+	+	+	+	+		+	
			c.[1513_1515del]	p.(Lys505del)	ALL: 0.0011%	−	−	−		Yes/HCM	
				(trans-acting variants)							
10	Frozen tissue (autopsy)	*TNNT2*	c.[275G>A]	p.(Arg92Gln)	−	Disease-causing	Deleterious	Probably damaging	35	Yes/HCM-LVNC	5
		LRG_431									
11	Frozen tissue (biopsy)	*TNNI3*	c.[509G>A]	p.(Arg170Gln)	−	Disease-causing	Deleterious	Possibly damaging	35	Yes/HCM	5
		LRG_432									
12	Sampling before death	*LMNA*	c.[568C>T]	p.(Arg190Trp)	−	Disease causing	Deleterious	Probably damaging	35	Yes/DCM –ARVC-LVNC	5
		LRG_254									
13	Fibroblasts collected before death	*TNNT2*	c.[421C>T]	p.(Arg141Trp)	−	Disease-causing	Deleterious	Probably damaging	33	Yes/HCM-DCM	5
		LRG_431									
14	Paraffin-embedded cardiac tissue	*TTR*	c.[118G>A]	p.(Val40Ile)	0	Disease-causing	Deleterious	Probably damaging	24.8	Yes/Amyloidosis	5
		LRG_416									
15	Sampling before death	*LMNA*	c.[610C>G]	p.(Leu204Val)	−	Disease-causing	Favour damaging	Possibly damaging	25.8	Yes/DCM	4
		LRG_254									

PSD: pre-symptomatic diagnosis; ARVC: arrhythmogenic right ventricular cardiomyopathy; HCM: hypertrophic cardiomyopathy; DCM: dilated cardiomyopathy; RCM: restrictive cardiomyopathy; LVNC: left ventricular non-compaction; MYH7: beta myosin heavy chain 7; TNNI3: troponin I; TNNT2: troponin T; DSP: desmoplakin; LMNA: lamin A/C; MYBPC3: myosin-binding protein C; TTR: transthyretin.

NM_000257.3 for *MYH7*, NM_000256.3 for *MYBPC3*, NM_001276347.1 for *TNNT2*, NM_000363.4 for *TNNI3* and NM_000432.3 for *MYL2*, NM_170707.2 for *LMNA*, NM_004572.3 for *PKP2*, NM_004415.2 for *DSP*, NM_001943.3 for *DSG2* and NM_000371.3 for *TTR*.

### 5.3 Impact of genetic results on the management of families

**Figure 2:**
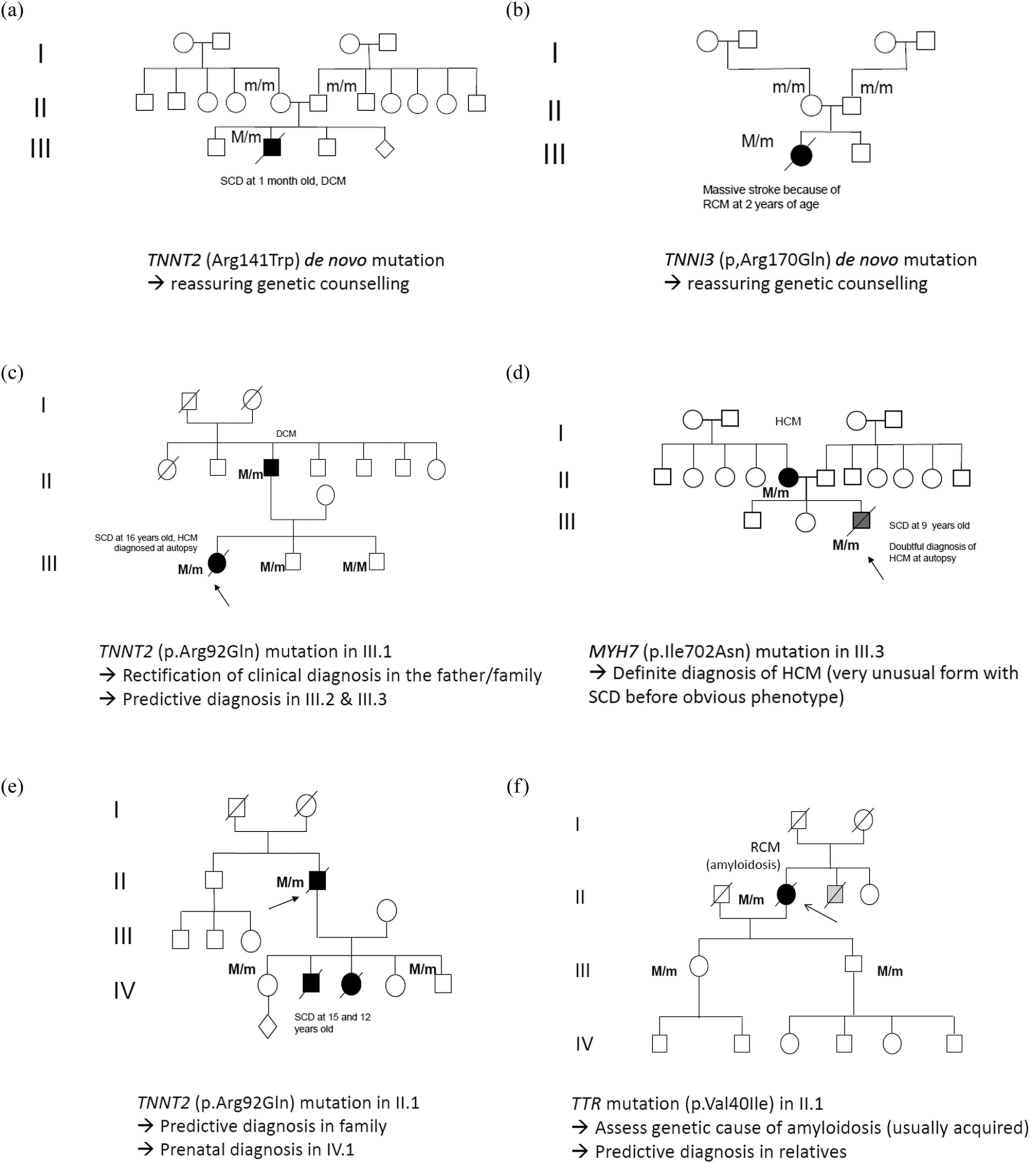
Selected pedigrees of patients with a causal variant and particular medical impact in the family.

The result of genetic testing was transmitted to the close relatives of the 15 deceased with a pathogenic causal variant, and various medical impacts were observed (Figure 2).

#### 5.3.1 Plan specific or early treatment based on precise subtype diagnosis

The identification of a given causal variant allowed in some cases the adjustment of the management of relatives. A causal variant in *LMNA* was identified in three patients (patient nos. 4, 12 and 15) whose relatives are therefore at higher risk of SCD (due to ventricular arrhythmia or conduction defect), and early use of a defibrillator and/or pacemaker should be considered in causal variant carriers of these families (with a lower threshold than used in patients with a similar cardiomyopathy but a different gene defect) [23]. Similarly, the *TNNT2* causal variant identified in patient nos. 8 and 10 is considered malignant (previously reported as such in the literature) [24], and the threshold for defibrillator implantation is also lower in relatives who carry the causal variant.

#### 5.3.2 *De novo* causal variant

In two families (patient no. 11 with massive stroke at 2 years of age revealing RCM; patient no. 13 with multiorgan failure at 1 month revealing DCM), further genetic analyses of the parents of the deceased indicated a *de novo* causal variant, since the variant was absent in the parents (blood leucocytes; Figures 2a and b). Therefore, molecular diagnosis in two cases allowed reassurance of the parents and siblings, since the risk of recurrence in the family was null or very low (theoretical risk of germline mosaicism).

#### 5.3.3 Revision of the clinical diagnosis

In one family (patient no. 10; Figure 2c) with a history of DCM in the father (LVEF 30% and normal LV thickness), SCD occurred in his daughter during a volleyball session (16 years of age), with no prior symptoms or diagnosis and, surprisingly, HCM was diagnosed at autopsy (wall thickness 28 mm). Post-mortem genetic analysis of frozen heart tissue obtained from the daughter identified a heterozygous causal variant of *TNNT2* gene and this variant was subsequently identified in the father. Therefore, the result of molecular testing definitely rectified the diagnosis of the cardiomyopathy subtype in the family, HCM and not DCM (the father probably exhibited an end-stage form of HCM mimicking DCM). In the case of patient no. 4 with a clinical diagnosis of ARVC, a causal variant in the *LMNA* gene was identified and also led to a rectification of diagnosis (biventricular cardiomyopathy or DCM due to laminopathy).

#### 5.3.4 Assessment of the diagnosis of cardiomyopathy at a preclinical stage with SCD

SCD occurred in patient no. 5 (aged 9 years with no prior symptoms or diagnoses) with only borderline diagnosis of HCM at autopsy (with relatively normal LV wall thickness of 0.7–1.2 cm but microscopic abnormalities fibrosis and myocyte disarray). A causal variant in the *MYH7* gene was identified in the child and also in the mother who exhibited apical HCM (Figure 2d). Molecular genetics was therefore able to elucidate the cause of SCD in the child and indicate a direct relation with the cardiomyopathy of the mother.

#### 5.3.5 Prenatal diagnosis

SCD occurred at 66 years of age in a man with HCM (Figure 2e). His family history also highlighted two children with SCD (at 12 and 15 years of age). Post-mortem genetic studies in the index patient identified a causal variant in the *TNNT2* gene, which was previously reported in other families with malignant HCM [7,24]. Predictive testing was proposed to relatives and the variant was present in two, including a woman who was later on pregnant and asked for prenatal diagnosis, which was agreed. Fortunately, the foetus did not carry the variant.

#### 5.3.6 Assessment of the genetic cause of cardiomyopathy that is usually acquired

Cardiac amyloidosis was diagnosed on myocardial biopsy in a 74-year-old woman (patient no. 14; Figure 2f) with severe heart failure, who rapidly died. The most prevalent cause of amyloidosis is acquired (usually light-chain deposition) but sometimes may be genetic. Genetic analysis was performed at the request of the family on paraffin-embedded tissues. Identification of a *TTR* causal variant led us to affirm the genetic origin of the disease, with a 50% risk in first-degree relatives of carrying the variant. In contrast with the initial counselling of the family, cardiac screening and predictive genetic screening were therefore recommended (a daughter and a son were subsequently identified as carriers of the causal variant).

#### 5.3.7 Predictive genetic testing and guidance of family screening and follow-up

In the usual context of delayed cardiac expression of cardiomyopathies, molecular diagnosis of deceased led to appropriate genetic counselling of relatives based on predictive testing (or pre-symptomatic testing), which was proposed to first-degree relatives in the 15 families with a causal variant. Early identification of causal variant carriers allowed close cardiac follow-up in order to deliver early and optimal treatment (including sports restriction, pharmacologic treatment and discussion about the use of a prophylactic defibrillator) and to reduce the risk of complications.

In the case of patient no. 10, the younger brother of the deceased daughter wanted to join the army. He underwent predictive testing, did not carry the causal variant and was, therefore, able to join the army and perform high-level physical activity without particular risk.

## 6 Discussion

We performed post-mortem genetic testing in 35 patients with SCD or other cardiovascular death and suspected cardiomyopathy. We identified a causal variant in 43% of these patients and various medical impacts of the results were observed in the families.

There is an increasing number of published reports of post-mortem genetic analyses [15,25,26,27] but usually in the context of SCD in young adults and normal autopsy [28,29,30]. Few post-mortem genetic studies have focused on autopsy in the case of suspected cardiomyopathy [16,17,18,19,20], and there has been no specific analysis of the clinical impact on the families.

Our report confirms the feasibility and significant yield of causal variant screening in this context. The technical feasibility of DNA extraction and quality control was good in our population, but this was expected since all analyses but one were performed using frozen fresh tissues (optimal for spleen or liver in our experience, slightly less for myocardium) or blood samples. This is in line with another report in the general field of post-mortem analyses [31]. Our results would have been probably worse in the case of more patients with paraffin-embedded tissues or fixed tissues, although the recent improved protocols also suggest feasibility in this situation [32]. The yield of causal variant screening was 43% in our cohort, which may be comparable to the figure of 30–60% in conventional testing in living patients with cardiomyopathy, depending on the precise subtype and sequencing technology [3,6]. In the context of post-mortem genetic analyses, a publication focused on suspected cardiomyopathies [16] obtained a yield of 22% (9 causal variants of 41 patients) with Sanger sequencing in a limited number of genes quite similar to those of the present report. In other recent publications using a large panel of genes and high-throughput sequencing technology, the yield of causal variant screening was 32–50% in a subgroup of patients with cardiomyopathy [18,19,20,26].

We observed that the medical impact of the genetic results was considerable in the families with a causal variant, with a clear added value as compared to prior suspicion of cardiomyopathy (based on autopsy or clinical data). Molecular results were used to make a diagnosis in a patient with uncertain or borderline cardiomyopathy on autopsy, illustrating a rare situation of early SCD at a preclinical stage of cardiomyopathy [4,5,33]. Molecular results rectified the cardiomyopathy subtype observed in other families, especially illustrating the difficult interpretation of the DCM phenotype, which might be the end-stage phase of another cardiomyopathy (such as HCM in one family) and lead to different cardiac follow-up and management of the relatives. In some families, molecular results led to the adjustment of treatment in relatives in the case of specific phenotype–genotype correlations, thus illustrating adjusted cardiac management (such as low threshold for prophylactic defibrillator implantation) in the presence of particular genes or causal variants responsible for a given cardiomyopathy [7,23,24]. Molecular results also led a family to ask for prenatal testing in a particular situation of a malignant causal variant and several cases of SCD. Finally, the molecular results enabled the offer of genetic counselling and predictive genetic testing to the first-degree relatives of all victims with a causal variant, which would not have been possible in most families, because there were no additional living relatives with a clear cardiomyopathy and therefore no additional index patients for primary causal variant screening. All these favourable medical issues illustrate the added value of post-mortem genetic analyses in the particular situation of suspected cardiomyopathy and show that these analyses provide further evidence for and strengthen the existing recommendations [11,12,13,34].

Our experience also underlines the complex ethical, legal and psychological issues associated with post-mortem genetic analyses [14,34,35]. To identify the referral person in the family after SCD, obtain written consent for genetic analyses and organise the information of the family about the genetic results, which was sometimes difficult and time-consuming. The legal framework for post-mortem genetic analyses may vary from country to country and could be clarified and improved in some countries, such as France. Post-traumatic stress in family members requires dedicated psychological support in most families. Close contact with forensic or medical and legal departments is required in order to provide tissues prepared in optimal conditions and then to transfer the material to the molecular genetics department.

All these various medical and nonmedical impacts or issues underline the need for close liaison between complementary expert teams and the utility of identifying multidisciplinary expert teams in that field as well as developing specific management at the national level, as recently suggested in European recommendations for the use of post-mortem genetic analyses [14].

## 7 Limitations

Our results may not apply to other causes of SCD, especially patients without suspected cardiomyopathy and with negative autopsy. Our results may also not apply to the use of large panels of genes with high-throughput sequencing technology, which may elucidate more causes of cardiovascular death and may improve the yield of mutation screening. Indeed, our present screening was not exhaustive since it was based on Sanger sequencing at the time of study. No further sequencing analyses on additional genes could be performed in this cohort due to insufficient sample quantity.

## 8 Conclusion

A causal variant was identified through post-mortem sequencing in nearly half of the patients with SCD or other cardiovascular death and suspected cardiomyopathy. The results had various medical impacts on families and enabled improved management of the families. Post-mortem molecular testing should be part of the management of family care after SCD or acute heart failure, even if cardiomyopathy is suspected at autopsy, since genetic findings provide additional important information useful for the management of relatives.
